# Linkage Between Hourly Precipitation Events and Atmospheric Temperature Changes over China during the Warm Season

**DOI:** 10.1038/srep22543

**Published:** 2016-03-02

**Authors:** Chiyuan Miao, Qiaohong Sun, Alistair G. L. Borthwick, Qingyun Duan

**Affiliations:** 1 State Key Laboratory of Earth Surface Processes and Resource Ecology, College of Global Change and Earth System Science, Beijing Normal University, Beijing 100875, China; 2 Joint Center for Global Change Studies, Beijing 100875, China; 3 School of Engineering, The University of Edinburgh, The King’s Buildings, Edinburgh EH9 3JL, UK

## Abstract

We investigated changes in the temporospatial features of hourly precipitation during the warm season over mainland China. The frequency and amount of hourly precipitation displayed latitudinal zonation, especially for light and moderate precipitation, which showed successive downward change over time in northeastern and southern China. Changes in the precipitation amount resulted mainly from changes in frequency rather than changes in intensity. We also evaluated the linkage between hourly precipitation and temperature variations and found that hourly precipitation extreme was more sensitive to temperature than other categories of precipitation. A strong dependency of hourly precipitation on temperature occurred at temperatures colder than the median daily temperature; in such cases, regression slopes were greater than the Clausius-Clapeyron (C-C) relation of 7% per degree Celsius. Regression slopes for 31.6%, 59.8%, 96.9%, and 99.1% of all stations were greater than 7% per degree Celsius for the 75th, 90th, 99th, and 99.9th percentiles for precipitation, respectively. The mean regression slopes within the 99.9th percentile of precipitation were three times the C-C rate. Hourly precipitation showed a strong negative relationship with daily maximum temperature and the diurnal temperature range at most stations, whereas the equivalent correlation for daily minimum temperature was weak.

Precipitation is a key component of the global water cycle and an active variable associated with atmospheric circulation^1^,^2^. A suite of analyses of changes in daily rainfall characteristics and extreme events has been released^3^,^4^,^5^; however, rainfall varies in a time-dependent fashion, even within a single precipitation event. Measurement of precipitation on a finer temporal scale, for example, hourly rainfall, better reflects the essential physical characteristics of precipitation and hence is important in many fields^6^. Changes in the characteristics of short-term precipitation events are of interest because of the potential hydrologic impact of these events, including flash floods, erosion, landslides, debris flows, and urban water-logging^7^,^8^. Precise precipitation analysis based on sub-daily temporal data is therefore essential to accurately determine rainfall intensity and assess the related societal impacts, and to understand the physical processes that drive precipitation intensity.

There has been an increase in research addressing the characteristics of precipitation measured at an hourly resolution. Dai *et al*. evaluated four different satellite datasets of hourly and 3-hourly precipitation events and found that the diurnal precipitation cycle at most low and mid latitudes derives predominantly from changes in precipitation frequency rather than changes in precipitation intensity^9^. Yu *et al*. used hourly precipitation data to show that diurnal variations in summer precipitation over China differ considerably between regions, and to document the relationship between rainfall duration and diurnal variation^10^,^11^. Li *et al*. used hourly data to address changes in duration-related regional precipitation characteristics across central eastern China^12^. Lastly, Zhang and Zhai, and Yu *et al*. have analyzed changes in the characteristics of hourly precipitation across eastern China over the past 40 years^6^,^8^.

A warmer atmosphere tends to hold more moisture and, within the context of global warming, induces extreme rainfall events with higher rainfall intensity^13^,^14^,^15^. The Clausius–Clapeyron (C-C) relation indicates that the rate of increase in atmospheric moisture-holding capacity is approximately 7% per degree Celsius of warming. Previous studies have reported that the intensity of extreme daily rainfall in a global climate model increases at the same rate^15^,^16^,^17^,^18^. However, the intensity of hourly precipitation has been demonstrated to exhibit a “super C-C” relationship with rises in temperature, featuring a rate of increase that exceeds 7% per degree Celsius of warming. Lenderink and van Meijgaard evaluated the records of hourly rainfall from a single station in De Bilt, Netherlands, and found that changes in hourly intensity generally increased at the 7% per degree Celsius rate anticipated by the C-C relation at temperatures below 10 °C, but at a rate greater than 7% per degree at higher temperatures^16^. Furthermore, linear regression between hourly extreme precipitation and daily mean temperature across the contiguous United States during the period 1950–2009 produced regression slopes greater than 7% at about 80% of the stations^19^.

Conversely, changes in precipitation affect temperature variations at both regional and larger scales^20^. Intuitively, the relationship between precipitation and surface temperature results in cooler conditions when precipitation occurs in summer and warmer conditions when precipitation occurs in winter^21^; however, some evidence indicates a strong relationship between precipitation deficits and the subsequent occurrence of hot extremes in a large fraction of the world^22^. Moreover, precipitation is normally closely correlated with cloudiness and soil moisture and thus impacts the minimum, maximum, and average temperatures, inevitably influencing the diurnal temperature range (DTR)^20^,^23^,^24^. However, few studies have addressed the relationship between temperature and hourly precipitation.

The climate over China plays an important role in the larger-scale Asian climate system. The East Asia monsoon, which dominates the climate in China, is largely regulated by the terrain characteristics of China: high in the west and low in the east. Owing to the remarkable topographic gradients and complexity of the landscape in China, the climate varies greatly over space and time^25^, and changes in mean precipitation vary with the seasons and regions. Regional differences in the characteristics of hourly precipitation are significant. Information on the temporospatial features of hourly precipitation across China would be meaningful for disaster evaluation and for understanding the physical mechanisms underlying precipitation. Moreover, testing the linkage between fine-scale precipitation (hourly) and temperature variation could yield an important climate simulation benchmark and provide insights into the possible future state of terrestrial energy and water budgets^26^. However, there is currently only limited research on these topics in the region. Therefore, the objectives of this work were to: (1) identify temporospatial changes in the characteristics of hourly precipitation over China; (2) quantify the rate of change of extreme hourly precipitation with temperature; and (3) evaluate the influence of hourly precipitation on temperature variations. Since the climate in China is dominated by the East Asia summer monsoon with summer precipitation accounting for about 70% of the annual rainfall, this study focused on the variations during the warm season (May–September).

## Results

### Temporospatial changes in hourly precipitation

**Figure 1:**
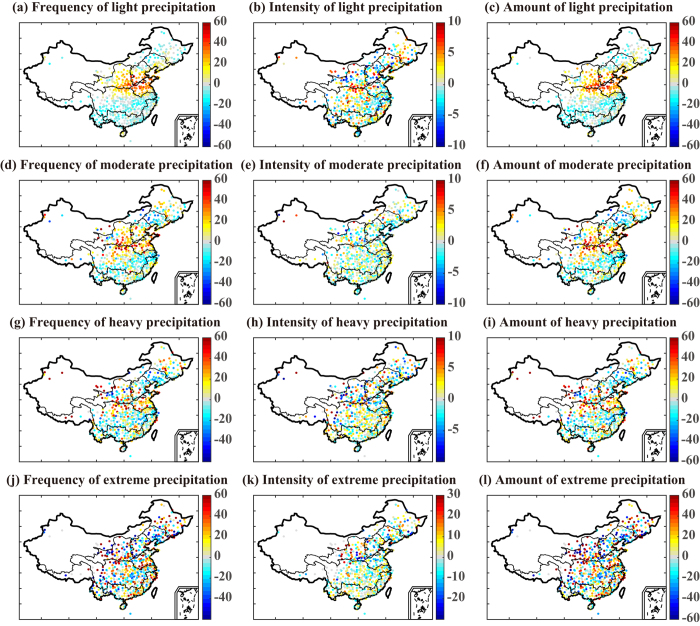
Percentage changes across mainland China in frequency, intensity, and total precipitation amount for hourly light, moderate, heavy, and extreme precipitation events between the periods 1991–2001 and 2002–2012. The maps were created using MATLAB (http://www.mathworks.com).

Figure 1 presents the changes in the frequency, intensity, and total amount of precipitation between two periods (1991–2001 and 2002–2012) for four classes of precipitation event: light (<5 mm/h), moderate (5–10 mm/h), heavy (10–20 mm/h), and extreme (>20 mm/h). Changes in the frequency and amount of light precipitation measured on an hourly basis showed latitudinal zonation (Fig. 1a,c). Stations around 30°N–40°N covering the Huaihe River basin, the Yellow River basin, the southern part of the Haihe River basin, and the northern part of the Yangtze River basin experienced large increases in the number of hourly light precipitation events after 2002, leading to an overall increase of more than 20% compared with the period 1991–2001. In contrast, after 2002, there were decreases in the total amount of light precipitation and the frequency of light precipitation events in northeastern and southern China, especially in the southern part of the Southwest River drainage basin. For moderate events, the changes in precipitation were similar and also showed regional differences by latitude. The locations with the largest increases in both frequency and total amount were concentrated in the northern section of the Yangtze River basin and the middle reaches of the Yellow River basin. In contrast, relatively large decreases were observed in the lower reaches of the Yangtze River basin and the northwestern part of the Southeast River drainage basin. For heavy precipitation events, zonal changes in frequency and amount were relatively weak. Increases were observed in the Huaihe River basin and the northern part of the Yangtze River basin, with about a 25% incremental rise in heavy rainfall amount. Hourly heavy precipitation decreased after 2002 in the Liaohe River basin and over a large part of southeastern China. For extreme precipitation, changes tended to be at the local rather than zonal level. In southern China, changes in extreme precipitation intensity were relatively weak. Large changes in the amount of extreme precipitation were observed in the Yellow River basin and the Songhua River basin, presumably resulting from the remarkable changes in both frequency and intensity of extreme precipitation in those regions (Fig. 1j,l). In addition, we investigated changes in frequency and intensity in extreme values by comparing the 5-year return values in 1991–2001 and 2002–2012 periods. Results showed the changes in frequency are statistically significant in the Huaihe River and Yellow River basins; The changes in intensity of wet events were most apparent in the Liaohe River and Haihe River basins (Fig. S1).

**Figure 2:**
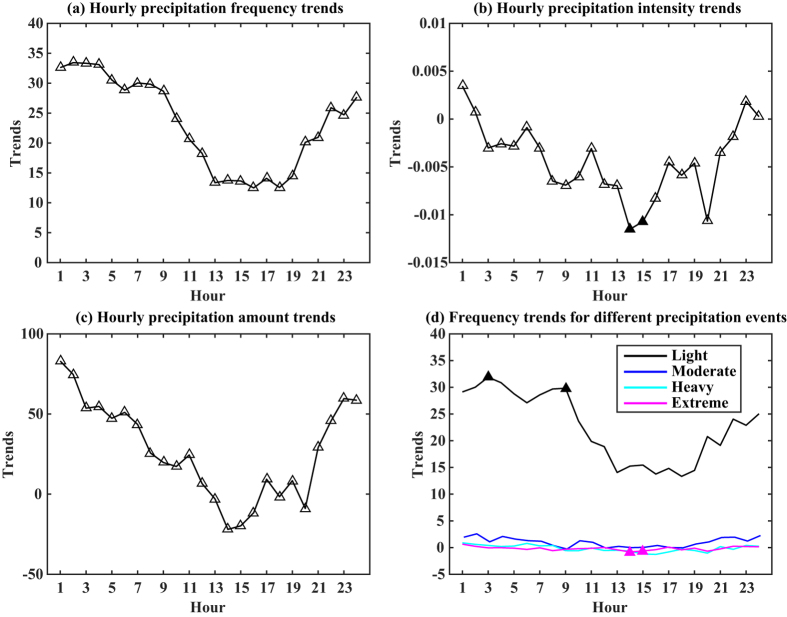
Trends in the diurnal cycle for precipitation frequency (**a**), intensity (**b**), and total amount (**c**), and for the frequency of different precipitation events (**d**) over China during the period 1991–2012. The rates of change corresponds to the slope of the linear regression, and positive (negative) values indicate increasing (decreasing) trends. The solid triangles indicate statistically significant changes at the *p* < 0.05 level.

Diurnal variations in precipitation provide information on the process of rain formation and its interaction with the local climate^11^. Two peaks in diurnal rainfall—one in the early morning and another in the late afternoon—have been identified over central eastern China^20^. However, this diurnal cycle of precipitation shows large spatial and seasonal variations^9^,^27^. Figure 2a shows an upward trend in the frequency of hourly wet events over China between 1991 and 2012, with positive rates of change for all hours throughout the day; the figure also shows that the rate of change was not the same at all times of day but that two distinct diurnal phases were evident. The rate of change in precipitation frequency was greater before noon than after noon [1300–1900 Local Standard Time (LST)]: the rates of change for these two phases were ~25-fold larger per annum and ~12-fold larger per annum, respectively. There was an overall downward trend in the intensity of precipitation for most times throughout the day, with positive rates of change occurring only around midnight LST (Fig. 2b). Combining the frequency and intensity changes for each local hour, the total amount of precipitation showed an upward trend for most hours, with the rate of change exceeding 40 mm per year between 2200 and 0700 (Fig. 2c). Downward trends were observed in the early afternoon (1300–1400) and at 1800 and 2000 LST. Hence, changes in the amount of precipitation resulted mainly from changes in frequency (at local time) rather than changes in intensity. In addition, we examined the change in precipitation frequency for the different precipitation classes. There were two distinct phases to the changes in the number of light precipitation events, similar to the phases in the overall frequency of precipitation (Fig. 2d) and the rate of change for light precipitation was greater than for the other classes. This indicates that changes in the overall frequency of precipitation resulted mainly from changes in the number of light precipitation events. At 0300 and 0900 LST, the rate of change for frequency of light precipitation reached statistical significance at the *p* < 0.05 level.

**Figure 3:**
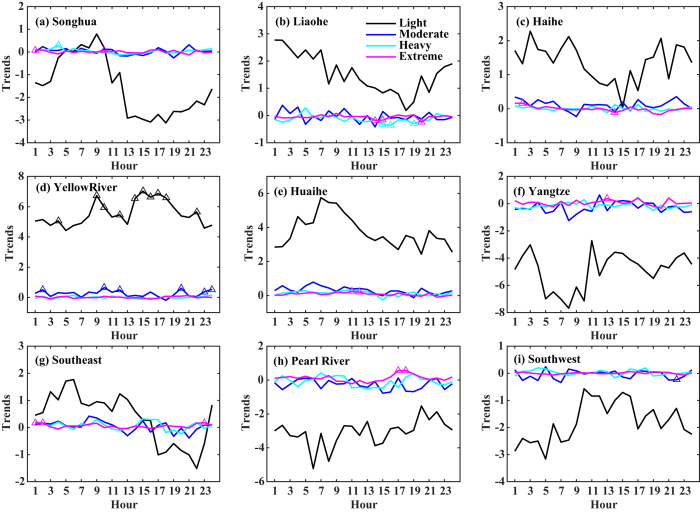
Trends in the diurnal cycle for the frequency of light (black line), moderate (blue line), heavy (cyan line), and extreme (magenta line) precipitation events in different river basins in mainland China during the period 1991–2012. The rate of change corresponds to the slope of the linear regression, and positive (negative) values indicate increasing (decreasing) trends. The triangles indicate statistically significant changes at the *p* < 0.05 level.

We similarly investigated trends in the diurnal frequency of hourly light, moderate, heavy, and extreme precipitation events for nine individual river basins between 1991 and 2012 (Fig. 3). The results show that the changes varied among regions. For all basins, the frequency of light events showed the greatest variability in rate of change across the day. Downward trends were observed in the Songhua River basin, the Yangtze River basin, and the Southwest River drainage basin. In the Songhua River basin, the most distinct changes were observed during the afternoon and night (1300–0000 LST), when decreases reached ~3-fold per annum. The Yangtze River basin showed the greatest downward trends in hourly light events, especially during the morning. In the Pearl River basin during the afternoon, the frequency of light events decreased but the frequency of extreme events increased. The Liaohe River, Haihe River, Yellow River, and Huaihe River basins showed upward trends in the frequency of light events for each of the 24 hours. The greatest changes were generally concentrated in the morning hours for the Liaohe River, Haihe River, and Huaihe River basins. The Yellow River basin showed a constant upward trend in the frequency of light events throughout the day, with increases of greater than 4-fold per annum for each of the 24 hours. The Southeast River drainage basin experienced an increase in the frequency of light events in the morning and late at night, but a decrease in the frequency of light events between 1700 and 2100 LST. All basins showed only slight variations in the frequency of moderate, heavy, and extreme events, similar to the pattern observed across the entire country (Fig. 2d).

### Interactions between atmospheric temperature and hourly precipitation

**Figure 4:**
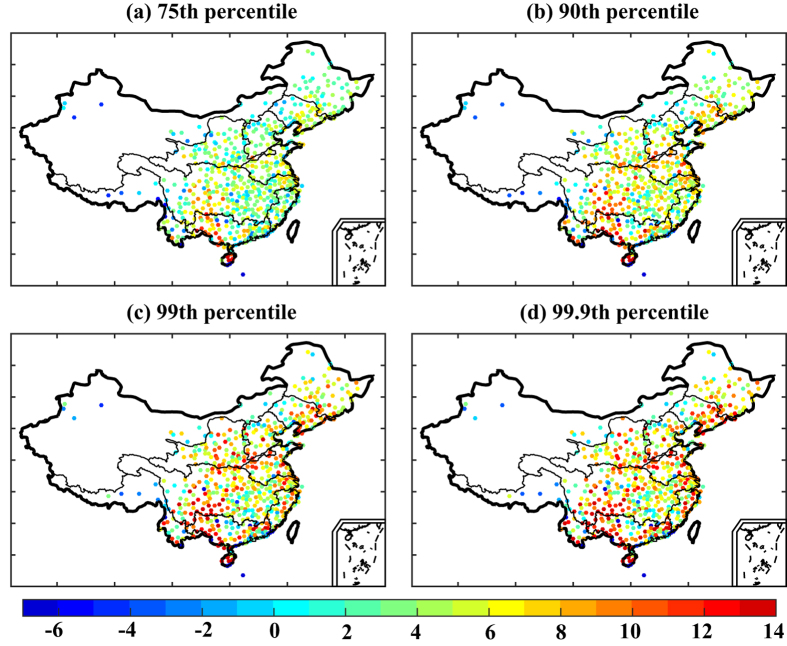
Spatial patterns across mainland China for the slopes of regressions between temperature and hourly precipitation (percentage change per degree Celsius). The maps were created using MATLAB (http://www.mathworks.com).

The annual rates of change for the 75th, 90th, 99th, and 99.9th percentiles of hourly precipitation during the period 1991–2012 period over China were estimated (Fig. S2), and the change rates of precipitation were uneven in different percentiles of precipitation and different regions. Previous studies revealed as the atmosphere warms, increased atmospheric water vapor content generally encourages an increase in extreme precipitation events^19^. Figure 4 shows the distribution of regression slopes for the relationship between hourly extreme precipitation amount and mean temperature for all stations, for the period 1991–2012. For the 75th percentile of precipitation, the regression slopes for most stations were lower than the C-C rate of 7% per degree Celsius, with the amount of rainfall generally increasing by around 4–6% per degree Celsius. Only a few stations scattered in the Yangtze River basin and the Pearl River basin had slopes greater than the C-C rate (Fig. 4a). For the 90th percentile of precipitation, the dependency of hourly precipitation extremes on temperature exceeded the C-C rate at about 30% of all stations; these stations were mainly located in the middle reaches of the Yangtze River and Pearl River basins, with a small number found in other river basins (Fig. 4b). For the 99th and 99.9th percentiles of precipitation, regression slopes greater than the C-C rate were found at 41.4% (240/580) and 47.2% (274/580) of all stations, respectively (Fig. 4c,d). These stations were concentrated in the Pearl River basin, the middle reaches of the Yangtze River and Yellow River basins, and the northern parts of the Huaihe River and Liaohe River basins. The corresponding regression slopes varied between 7% and 14% per degree Celsius. Overall, the regression slopes increased as the percentile of hourly precipitation increased, indicating that precipitation extremes were more sensitive to changes in temperature.

**Figure 5:**
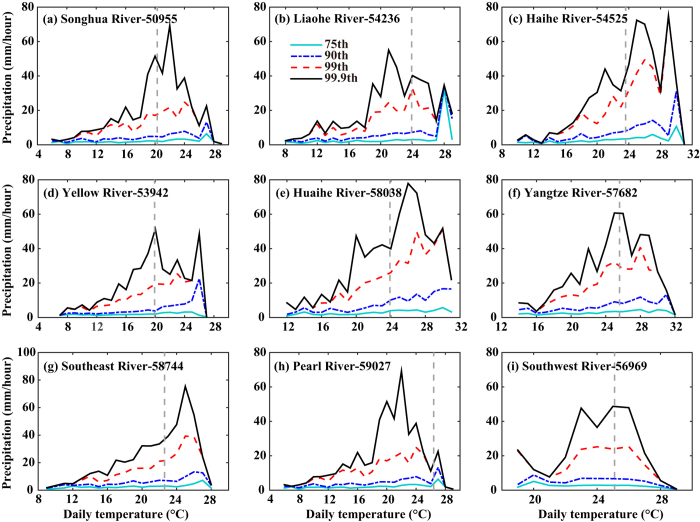
The relationship between hourly precipitation and temperature for nine stations, one in each of nine river basins in mainland China. The colored lines indicate the different percentiles of precipitation: 75th percentile (light blue solid line), 90th percentile (dark blue dashed line), 99th percentile (red dashed line) and 99.9th percentile (black solid line). The gray dashed lines indicate the median daily temperature and the numbers refer to the station index number.

**Figure 6:**
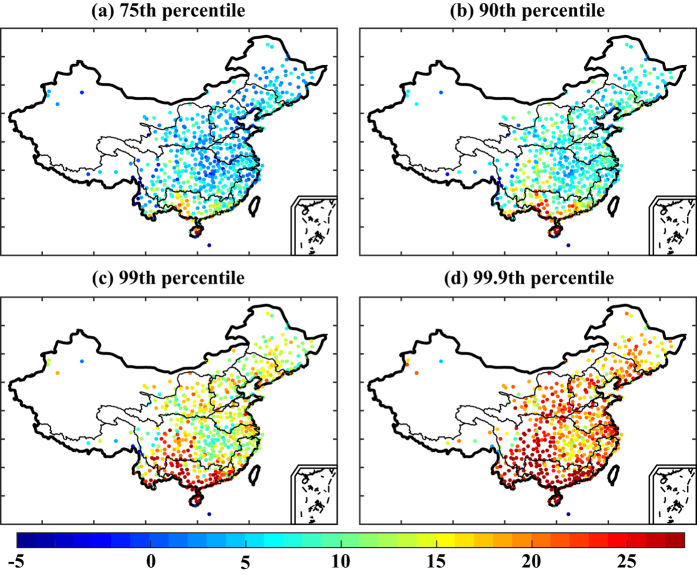
Spatial patterns across mainland China for the slopes of regressions between hourly precipitation and daily temperature at temperatures colder than the median daily temperature (percentage change per degree Celsius). The maps were created using MATLAB (http://www.mathworks.com).

Figure 5 shows the relationships between temperature and precipitation obtained from nine randomly selected stations, one in each of the nine river basins. The 75th, 90th, 99th, and 99.9th percentiles of precipitation intensity generally increased as temperatures increased, which suggests strong temperature dependency. However, a reduction in precipitation intensity was found when temperatures exceeded a higher temperature threshold, suggesting that the relationship between precipitation and temperature does not follow the C-C relationship. We therefore plotted the regression slopes for hourly precipitation and daily temperatures below the median daily temperature across mainland China (Fig. 6) and found the regression slopes to be greater than those shown in Fig. 4. For the 75th, 90th, 99th, and 99.9th percentiles of precipitation, 31.6%, 59.8%, 97.0%, and 99.1% of all stations had regression slopes greater than 7% per degree Celsius. The mean regression slopes were greater than the C-C rate for the 90th (8.9% per degree Celsius), 99th (16.0% per degree Celsius), and 99.9th (22.5% per degree Celsius) percentiles of precipitation, with the latter being approximately three times the C-C rate. Overall, the slope values were higher than those shown in Fig. 4, indicative of a decrease in precipitation at temperatures above the median daily temperature.

**Figure 7:**
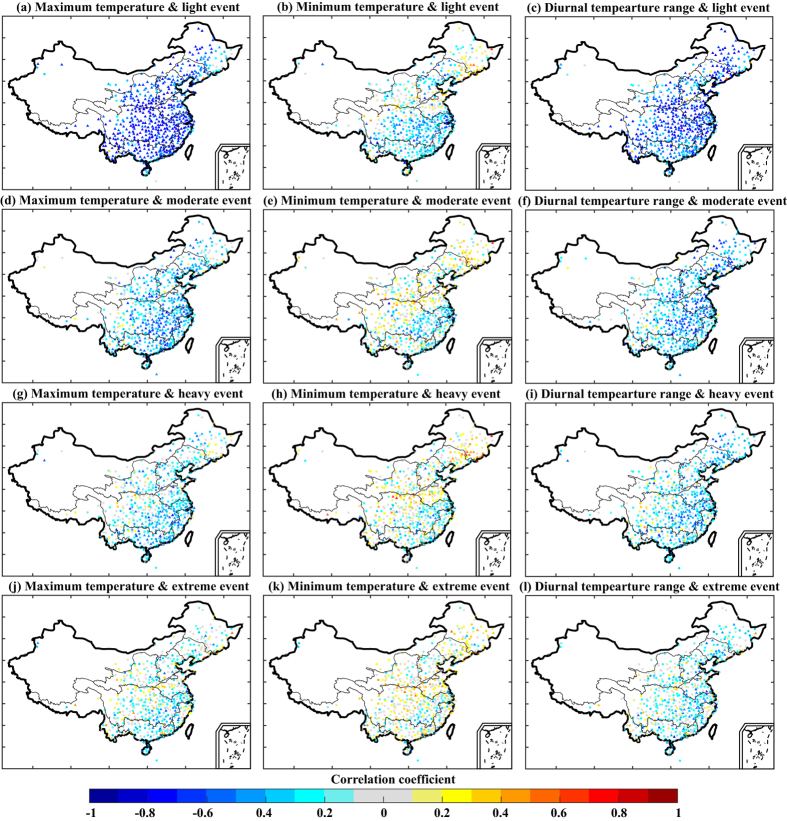
Spatial patterns of the correlations between temperature variations and the frequency of hourly light, moderate, heavy, and extreme precipitation events. The triangles indicate correlation coefficients that were statistically significant at the *p* < 0.05 level. The maps were created using MATLAB (http://www.mathworks.com).

Precipitation is associated with cloudiness and soil moisture and thus can be strongly correlated with the DTR. As shown in Fig. 7, the daily maximum temperature (TX) and frequency of light precipitation events exhibited a strong negative correlation (correlation coefficients below −0.6) over large regions for the period 1991–2012; correlations between the TX and other categories of precipitation event were weaker. The negative correlations for all four categories of precipitation event were stronger in southeastern China than in other regions. The correlations between daily minimum temperature (TN) and precipitation were weaker and also showed regional dependency. Stations located in the Yangtze River basin, Southeast River drainage basin, and parts of the Pearl River and Haihe River basins exhibited a negative relationship between TN and light precipitation, whereas weak positive correlations were found in northern China in the regions of the Huaihe River basin, the Yellow River basin, and the Songhua River basin. With increasing precipitation intensity, negative correlations decreased and positive correlations became more numerous. Only a few stations scattered in the lower Yangtze River basin showed a negative relationship between TN and precipitation for all four categories of precipitation. Changes in the DTR are sensitive to variations in TX and TN. Overall, the relationship between the DTR and hourly precipitation was consistent with that for TX and hourly precipitation; the frequency of hourly light events showed the strongest negative correlations with the DTR compared with the other precipitation categories, with the correlations for light precipitation being statistically significant at the *p* < 0.05 level for most stations . Our findings indicate that the frequency of hourly light precipitation has a range of effects on temperature variations.

**Figure 8:**
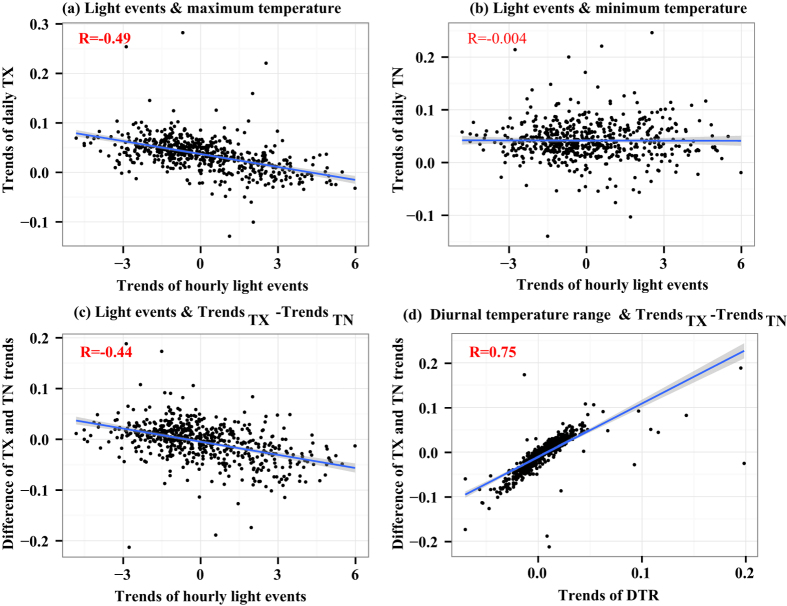
Scatter plots of data from 580 stations across mainland China for the trends in the frequency of light precipitation events and (**a**) daily maximum temperature (TX), (**b**) daily minimum temperature (TN), and (**c**) the difference between the trends in TX and TN. Panel (**d**) plots the difference between the trends in TX and TN and trends in the diurnal temperature range (DTR). Grey shading indicates the 95% confidence intervals. Correlation coefficient are shown in red in the upper left of each panel, with bold font indicating a statistically significant correlation at the *p* < 0.05 level.

We analyzed trends in the TX, TN, and the DTR and their association with the frequency of hourly light precipitation events at 580 stations across China for the period 1991–2012. Trends in the TX varied by approximately −0.05 °C per year to 0.1 °C per year (Fig. 8a). About half of the sites showed downward trends in hourly light precipitation events over the period 1991–2012, with upward trends in the corresponding maximum temperatures. Trends in TX related significantly to trends in the frequency of hourly light precipitation. For TN, upward trends of approximately 0 °C per year to 0.1 °C per year were observed at most stations (Fig. 8b). However, TN did not appear to be correlated with the frequency of light precipitation events, indicating that the changes in TN were not sensitive to precipitation change. The rates of change in TX and TN were not consistent at each station, with differences in trends varying from −0.1 °C per year to 0.1 °C per year. The relationship between these differences and trends in the frequency of light precipitation events resembled the relationship between trends in TX and light precipitation events. Approximately half of the stations exhibited positive differences, resulting in an increase in the DTR. Changes in the DTR were linearly dependent on the differences between the trends in TX and the trends in TN (Fig. 8d).

## Discussion

The frequency and magnitude of precipitation measured on an hourly basis over mainland China showed latitudinal zonation with three sequential areas apparent, involving the northeastern, northern, and southern regions of the country. This was particularly evident for light and moderate precipitation events, which showed downward trends over northeastern and southern China. These results are consistent with similar changes obtained from daily measures of precipitation by Wu *et al*., who attributed rain reduction to lower tropospheric temperatures and precipitation^28^. Previous studies have demonstrated that the diurnal variation in rainfall across China shows considerable regional fluctuations. Differences in the annual rates of change for daytime and nighttime precipitation could partly account for changes in diurnal variation. For instance, precipitation peaks in the late afternoon have previously been documented over southern and northeastern China^29^; however, we observed downward trends in the frequency of precipitation in the Songhua River basin and Southeast River drainage basin in the late afternoon and upward trends during the morning, weakening the diurnal variation. In addition, changes in hourly heavy and extreme precipitation were unevenly distributed, indicating sensitivity to location. Short-duration extreme precipitation was most likely associated with terrain or terrain-induced local circulation, such as local mountain–valley wind circulation^8^,^30^.

Temperature and precipitation are two of the most important climate variables, and understanding their relationship is important for identifying precipitation-forming processes and for weather and climate forecasting^31^. Our investigation into the dependency of hourly precipitation on daily mean temperature in the warm season showed that the regression slopes at most stations were approximately 4–6% per degree Celsius for the 75th percentile of precipitation and that only 41% (240/580) and 47% (274/580) of all stations showed regression slopes slightly greater than the C-C rate of 7% per degree Celsius for the 99th and 99.9th percentiles of precipitation, respectively. However, a stronger dependency of hourly precipitation on temperature occurred at temperatures colder than the median daily temperature, with 32%, 60%, 97%, and 99% of all stations exhibiting regression slopes greater than the C-C rate for the 75th, 90th, 99th and 99.9th percentiles of precipitation, respectively. The mean regression slope for the 99.9th percentile was three times the C-C rate. The results revealed that, at most stations, rainfall intensity increased with temperature up to a maximum and then decreased with temperature beyond that maximum. Precipitation formation involves processes acting at different spatial and temporal scales, such as large-scale atmospheric dynamics and meso-scale convection^17^. In China, compared with stratiform precipitation (such as larger-scale frontal precipitation), convective precipitation related to vigorous overturning tends to occur on warmer days and promotes heavier short-term rainfall. Stratiform events have no pronounced temperature dependence but convective events taken as a whole produce an increase in intensity of extreme events that exceeds the C-C rate^32^. Furthermore, in summertime, the tropical cyclone produces significant amounts of extreme rainfall in most coastal regions. These factors can partly explain the stronger dependency of hourly extreme precipitation (99.9th percentile) on temperature that was observed at most stations. However, our observation of a decrease in precipitation at high temperatures is contrary to the finding by Lenderink and Meijgaard that the dependency of hourly precipitation extremes on daily temperature was approximately twice the C-C rate for temperatures above 10 °C. Different geographical regions and seasons affect the relationship between temperature and precipitation^33^, so the appearance of a decrease in precipitation at high temperatures may be related to China’s specific geographical features. In China, the climate is generally controlled by complex large-scale atmospheric circulation systems. For instance, the moisture supply in China is mainly dominated by the western Pacific subtropical high and the East Asia summer monsoon, especially in summertime^34^,^35^, with consequent effects on both temperature and precipitation. High temperatures and a lack of rain are the predominant conditions under the western Pacific subtropical high during the warm season. Furthermore, Utsumi *et al*. raised the possibility that the duration of wet events may be related to the relationship between temperature and precipitation. They showed that a decrease in the duration of precipitation events can explain the decrease in extreme daily precipitation intensity at high temperatures^36^. Inconsistencies remain between the precipitation–temperature relationships measured at the global scale compared with the regional scale. With global warming, it might be expected that more extreme precipitation may occur at both regional and global levels^37^. However, the findings of our study suggest that precipitation may in fact decrease after the temperature exceeds a threshold value (Fig. 5). The combined effect of higher temperatures and less precipitation could produce heat waves and droughts, and thus challenge societal development.

Our results showed a strong correlation between the DTR and hourly light precipitation. The DTR is by nature related to changes in TX and TN). Whereas TN is strongly dependent on net longwave radiation, TX is strongly controlled by surface solar heating and the partitioning of sensible and latent heat fluxes. Previous studies have revealed cloud, precipitation, and soil moisture as the main factors affecting variations in the DTR. Compared with clear-sky days, days with precipitation (often accompanied by cloud cover) affect TX during the daytime and TN at nighttime owing to the influence on surface solar heating and upward longwave radiation^38^,^39^. Furthermore, precipitation can increase the soil moisture content, which in turn contributes to changes in the DTR. Verdecch *et al*. revealed that in areas where soil moisture increases owing to an increase in precipitation there is a positive change in latent heat flux and a decrease in sensible heat flux^40^; consequently, in areas with increased soil moisture, the increase in TX is smaller than the increase in TN, thereby causing a decrease in the DTR. In our study, hourly precipitation was significantly correlated with TX and the DTR at most stations, indicating that precipitation affects the DTR. In the warm season, short-term strong rainfall events, such as convective precipitation, were more sensitive to the changes in temperature. Overall, the frequency of hourly light precipitation had the strongest relationship with the DTR. Moreover, the correlation between precipitation and TN was weak. The rate of change in TX was generally inversely related to the rate of change of hourly precipitation frequency, especially for light precipitation events. It is likely that this relationship was primarily driven by reductions in solar heating owing to cloud cover and by increases in surface latent heat flux owing to increased surface wetness from precipitation^41^. However, the correlation between TX and precipitation frequency gradually weakened as hourly rainfall intensity increased from light to extreme. During the warm season, convective precipitation related to vigorous overturning can promote heavy rainfall over a short period of time. For convective precipitation, the maximum rainfall rate and peak intensity occur in the late afternoon^42^, which is generally later in the day than the time at which TX occurs. This partly explains the weaker relationship between TX and hourly heavy and extreme precipitation. Whereas Zhou *et al*. found a stronger relationship between increased precipitation and trends in TN and the DTR^20^, we have found a distinctive relationship between warm-season precipitation changes and trends in TX and the DTR. Other mechanisms that may contribute to changes in the DTR, such as clouds, aerosols, and land cover, should be explored in future studies.

In this study, we used gauge observations of precipitation at a fine (hourly) temporal resolution. However, owing to a lack of available data, our study used limited data and relatively short time periods and this may have restricted the results. Furthermore, gauge measurements do not provide complete areal coverage and are not available over unpopulated land areas, especially in western China. Recently, some satellite-derived datasets that provide sub-daily precipitation measurements and more spatially homogeneous and temporally complete coverage of large areas of the globe have become available. These include the Tropical Rainfall Measuring Mission (TRMM)^43^, the Precipitation Estimation from Remotely Sensed Information using Artificial Neural Networks (PERSIANN)^44^,^45^, and Climate Prediction Center morphing technique (CMORPH) products^46^. Moreover, the Global Precipitation Measurement (GPM) Core Observatory was launched in early 2014 to explore global precipitation characteristics in more detail^47^, and a high-resolution and advanced Integrated Multi-satellitE Retrievals for GPM (IMERG) dataset was recently released. The increased temporal and spatial resolution provided by these satellite observations offers great potential for improving precipitation measurements to meet the various scientific and societal needs.

## Data and Methods

### Data

**Figure 9:**
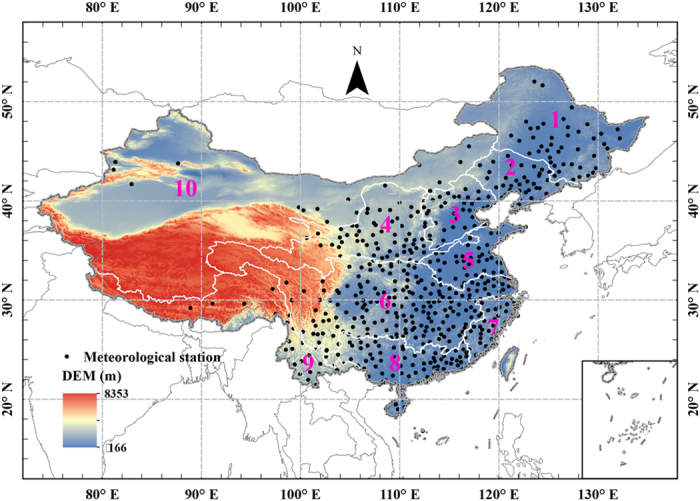
Location of the study area and distribution of the selected meteorological stations across mainland China: 1, Songhua River; 2, Liaohe River; 3, Haihe River; 4, Yellow River; 5, Huaihe River; 6, Yangtze River; 7, Southeast River drainage; 8, Pearl River; 9, Southwest River drainage; 10, Northwest River drainage. The map was created using ESRI ArcGIS 10.0 (http://www.esri.com/software/arcgis).

The dataset of hourly rainfall was obtained from the National Meteorological Information Center of the China Meteorological Administration, which maintains rain-gauge records from more than 2400 stations across China. Data on the daily mean temperatures, maximum temperatures, and minimum temperatures from 824 stations were collected from the SURF_CLI_CHN_MUL_DAY_V3.0 dataset, which was downloaded from the China Meteorological Data Sharing Service System (http://cdc.nmic.cn/home.do). The full dataset was subject to strict quality control and homogenized according to the methods described by Xu *et al*.^48^; 580 stations providing synchronous hourly precipitation and temperature measurements were selected for this research (Fig. 9).

### Analysis

To detect the changes in precipitation, it was necessary to examine variations both in mean precipitation and in precipitation at different intensities. We divided all hourly precipitation events across China into four categories: light (<5 mm/h), moderate (5–10 mm/h), heavy (10–20 mm/h), and extreme (>20 mm/h)^8^,^49^. We compared the changes in mean precipitation amount, intensity, and frequency in these four categories between the periods 1991–2001 and 2002–2012. We focused on each wet event, defined as hourly precipitation greater than 0.1 mm. To better understand the hydrologic processes, we also estimated changes in temperature and precipitation in the different river basins separately (Fig. 9). These data were used to investigate temporal changes in precipitation. Owing to the very few stations distributed in the Northwest River drainage basin, results for this basin were excluded. We investigated trends in the frequency of light, moderate, heavy, and extreme precipitation events over nine different river basins.

Previous studies have noted that hourly extreme precipitation events show increases at more than the 7% C-C rate in a variety of regions^17^. The present study utilizes the method proposed by Mishra *et al*.^19^ to investigate the relationship between mean daily temperature and hourly extreme precipitation events. For each station, all hourly precipitation events and the corresponding daily mean temperatures were extracted to calculate the relationships over the period 1991–2012. Hourly precipitation data were divided into bins of 1 °C width based on the daily mean temperature and, for each temperature bin, the 75th, 90th, 99th, and 99.9th percentiles of hourly precipitation were estimated. We used regression analysis to plot the logarithm of these hourly precipitation percentiles against the mean temperature to estimate the percentage change in hourly precipitation with temperature. Panthou *et al*. summarized the various relationships between precipitation and temperature as follows: (i) rainfall intensity increases monotonically with temperature, (ii) rainfall intensity decreases monotonically with temperature, (iii) rainfall intensity increases with temperature and levels off at high temperature (upper limit structure), and (iv) rainfall intensity increases with temperature and decreases after reaching a maximum (peak-like structure)^33^. We selected nine stations at random, one from each of the nine river basins, to examine the type of the relationship between temperature and precipitation. Noting that Isaac and Stuart found inconsistencies in the precipitation occurring at temperatures lower and higher than the median temperature^31^, we took the median daily temperature at each station as the threshold and analyzed the relationship between hourly precipitation and daily temperatures below and above this threshold.

Previous studies have also demonstrated that precipitation affects the mean, maximum, and minimum temperatures, and hence the DTR^23^,^41^. We utilized the Pearson correlation coefficient to estimate the correlation between hourly precipitation and temperature variations, and linear regression to calculate the rate of change of these variables.

## Additional Information

**How to cite this article**: Miao, C. *et al*. Linkage Between Hourly Precipitation Events and Atmospheric Temperature Changes over China during the Warm Season. *Sci. Rep*. **6**, 22543; doi: 10.1038/srep22543 (2016).

## Supplementary Material

Supplementary Information
