# Supplementary material for: Linkage Between Hourly Precipitation Events and Atmospheric Temperature Changes over China during the Warm Season
Source: Sci Rep. 2016 Mar 2;6:22543. doi: 10.1038/srep22543 (PMC4773837; doi:10.1038/srep22543)
Supplement: Supplementary Information [file srep22543-s1.doc]

**Linkage Between Hourly Precipitation Events and Atmospheric Temperature Changes over** **China during the Warm Season**

Chiyuan Miao^1,2^, Qiaohong Sun^1,2^, Alistair G.L. Borthwick^3^, Qingyun Duan^1,2^

^1^ State Key Laboratory of Earth Surface Processes and Resource Ecology, College of Global Change and Earth System Science, Beijing Normal University, Beijing 100875, China

^2^ Joint Center for Global Change Studies, Beijing 100875, China

^3^School of Engineering, The University of Edinburgh, The King’s Buildings, Edinburgh EH9 3JL, U.K.

*Corresponding authors:*

Chiyuan Miao, College of Global Change and Earth System Science, Beijing Normal University, Beijing 100875, China.

E-mail: miaocy@vip.sina.com; Tel.: +86-10-58804191; Fax: +86-10-58804191.

*Co-authors:*

Qiaohong Sun, College of Global Change and Earth System Science, Beijing Normal University, Beijing 100875, China ([88795760454@sina.com](mailto:88795760454@sina.com))

Alistair G.L. Borthwick, School of Engineering, The University of Edinburgh, The King’s Buildings, Edinburgh EH9 3JL, U.K. ([Alistair.Borthwick@ed.ac.uk](mailto:Alistair.Borthwick@ed.ac.uk))

Qingyun Duan, College of Global Change and Earth System Science, Beijing Normal University, Beijing 100875, China ([qyduan@bnu.edu.cn](mailto:qyduan@bnu.edu.cn))

**Discussion**

In addition to the main analysis we calculated and compared the 5-year return values for the frequency and intensity of wet events for two periods (1991 – 2001 and 2002 – 2012) over the nine river basins by referencing the methods in Kharin and Zwiers[^49^](#_ENREF_1). Firstly, the annual frequency and intensity were calculated for each river basin. Then, the extreme value distribution was fitted to the raw annual-values data and the corresponding 5-year return values were obtained by inverting the fitted extreme value distribution for the two periods. The results of the Kolmogorov Smirnov (K-S) test indicate that the extreme value distribution provides a reasonable description of the behavior of the frequency and intensity of wet events. The 80% confidence intervals estimated by bootstrapping are shown in Figure S1. The results displayed in Figure S1 are generally consistent with those in Figure 1 in the section “Temporospatial changes in hourly precipitation”. In the Huaihe River and Yellow River basins, the 5-year return values indicate that the frequency of wet events was greater during the 2002 – 2012 period than during the 1991 – 2001 period; the confidence intervals for 2002 – 2012 generally do not overlap with those for 1991 – 2001 for the Huaihe River and Yellow River basins, indicating that the changes in frequency are statistically significant. For the intensity of wet events, changes were most apparent in the Liaohe River and Haihe River basins.


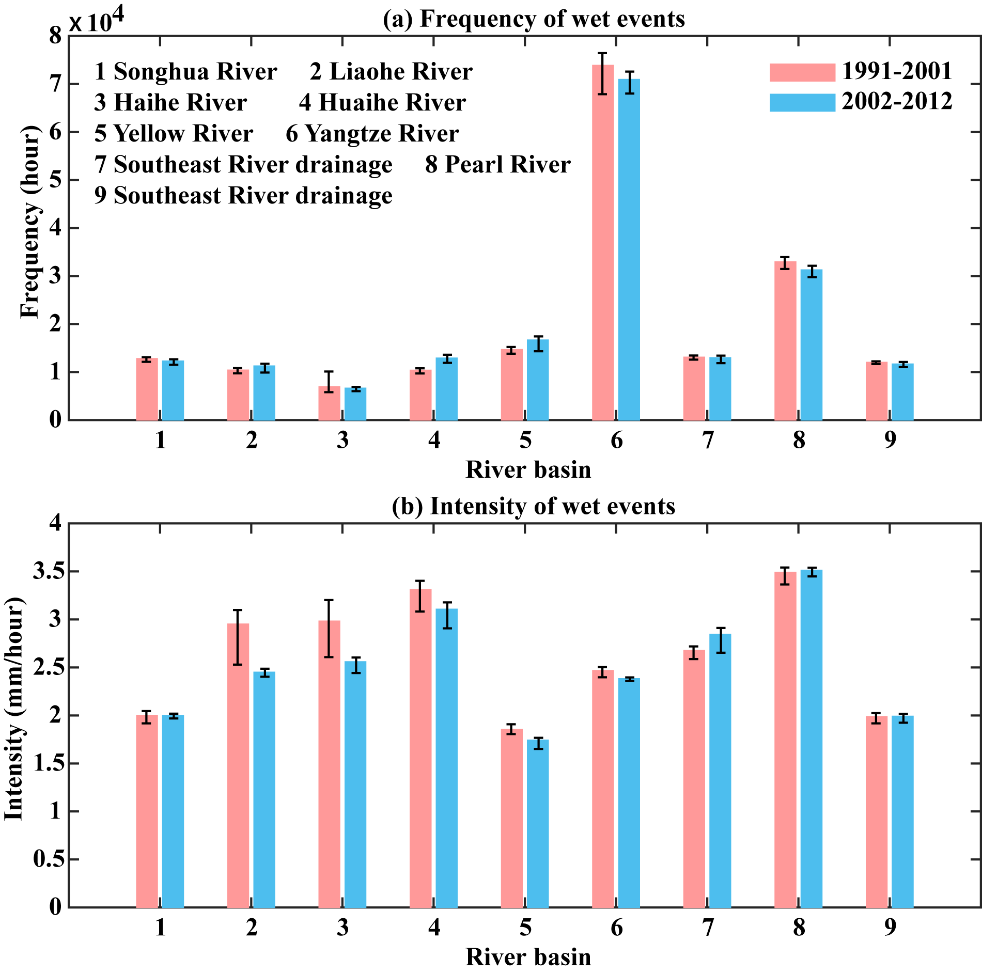


**Fig. S1.** 5-year return values for the frequency (a) and intensity (b) of wet events in 1991– 2001 and 2002 – 2012 for nine river basins, calculated from the raw data. The solid lines indicate the corresponding 80% confidence intervals estimated by bootstrapping.


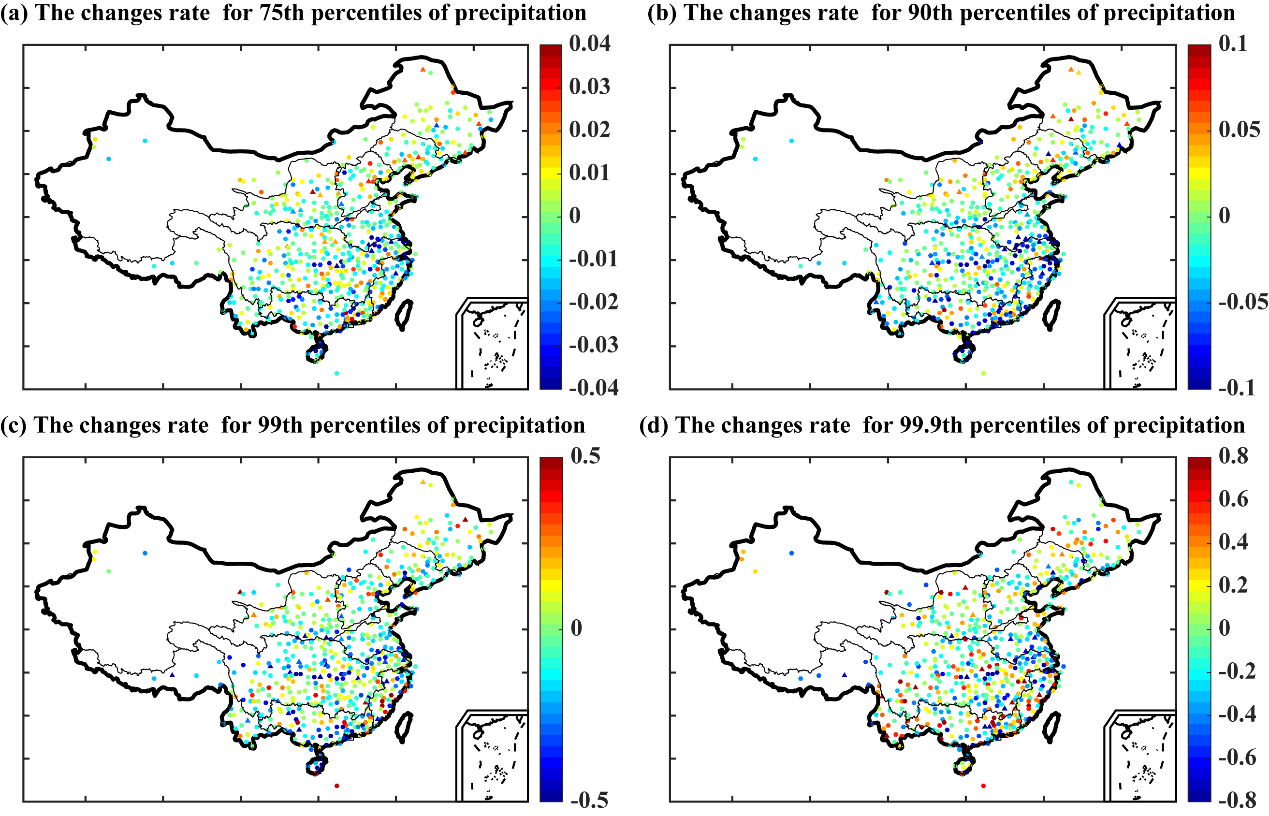


**Fig. S2.** Spatial patterns of the annual rates of change (mm/yr) for the 75th, 90th, 99th, and 99.9th percentiles of hourly precipitation during the period 1991 – 2012 period. The triangles indicate rates of change that were statistically significant at the *p* < 0.05 level. The maps were created using MATLAB (http://www.mathworks.com).
